# Supplementary material for: In vivo gastrointestinal drug-release monitoring through second near-infrared window fluorescent bioimaging with orally delivered microcarriers
Source: Nat Commun. 2017 Mar 10;8:14702. doi: 10.1038/ncomms14702 (PMC5353702; doi:10.1038/ncomms14702)
Supplement: Supplementary Information — Supplementary Figures, Supplementary Tables, Supplementary Methods and Supplementary References [file ncomms14702-s1.pdf]

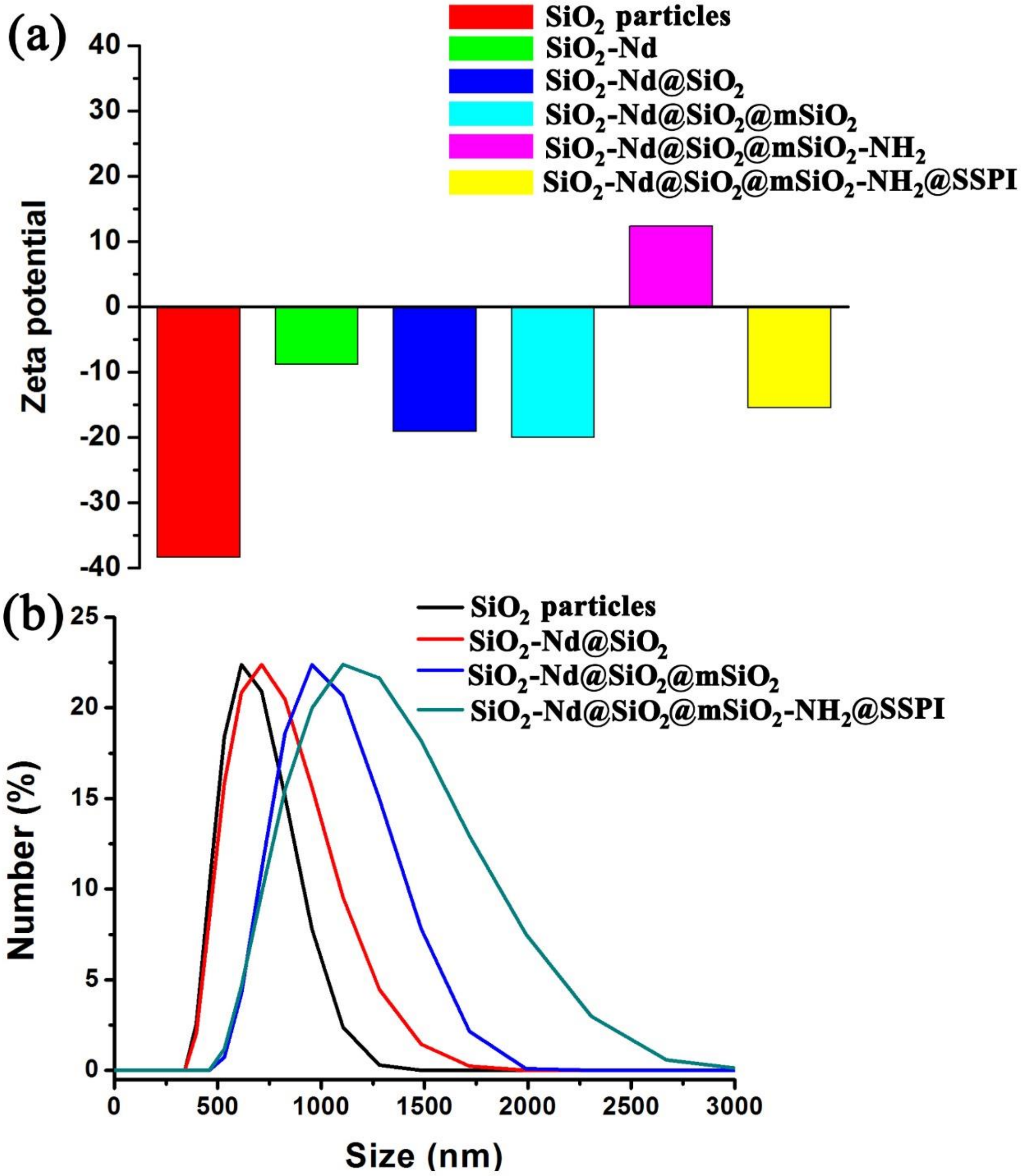

Supplementary Fig. 1. Hydrodynamic diameter and Zeta potential of the as-synthesized microcarriers (**a**) DLS data showing the hydrodynamic diameter of SiO_2 particles (614 nm, PDI = 0.023), SiO_2-Nd@SiO_2 (709 nm, PDI = 0.082), SiO_2-Nd@SiO_2@mSiO_2 (1020 nm, PDI = 0.057) and SiO_2-Nd@SiO_2@mSiO_2-NH_2@SSPI (1130 nm, PDI = 0.26). PDI is short for polydispersity index. (**b**) Zeta potential of SiO_2 particles (- 38.4 mV), SiO_2-Nd (- 8.8 mV), SiO_2-Nd@SiO_2 (- 19.1 mV), SiO_2-Nd@SiO_2@mSiO_2 (- 20.0 mV), SiO_2-Nd@SiO_2@mSiO_2-NH_2 (+ 12.4 mV) and SiO_2-Nd@SiO_2@mSiO_2-NH_2@SSPI (- 15.4 mV) at pH 7 buffer solution.

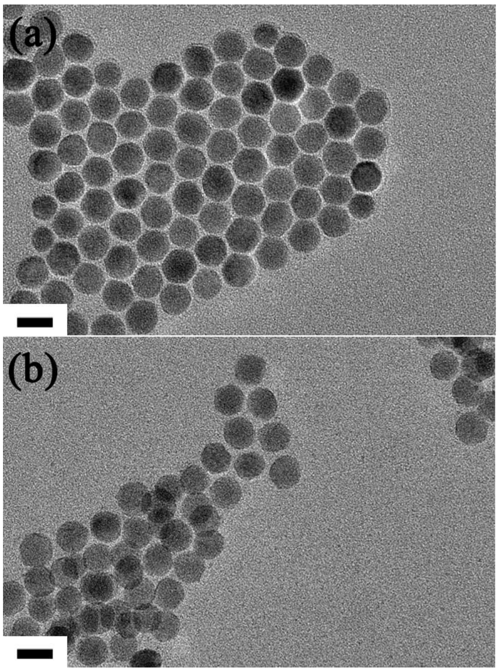

Supplementary Fig. 2. TEM images of as-synthesized DCNPs (**a**) TEM images of $NaGdF_4$:5%Nd@$NaGdF_4$ DCNPs. (**b**) TEM images of water-soluble $NaGdF_4$:5%Nd@$NaGdF_4$ DCNPs. Scale bar = 20 nm.

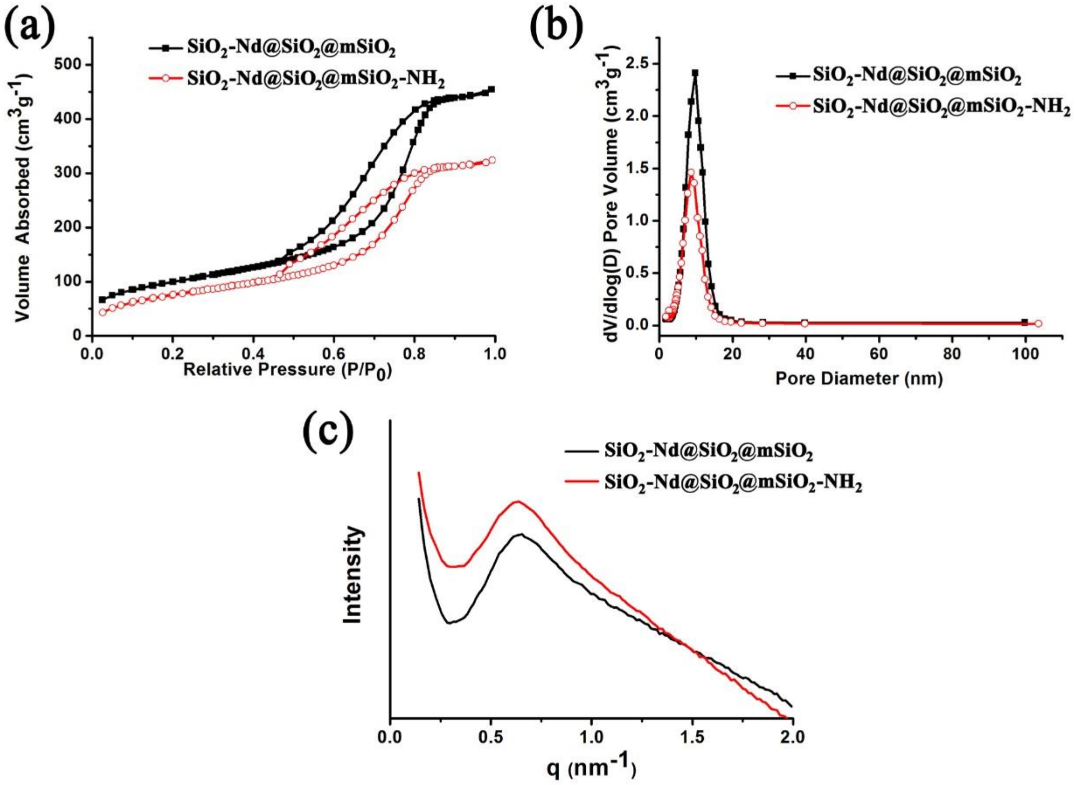

Supplementary Fig. 3. The characterization of mesopores on the microcarriers Nitrogen adsorption-desorption isotherms (**a**), pore size distribution (**b**) and SAXS patterns (**c**) of the SiO_2-Nd@SiO_2@mSiO_2 and SiO_2-Nd@SiO_2@mSiO_2-NH_2 particles respectively.

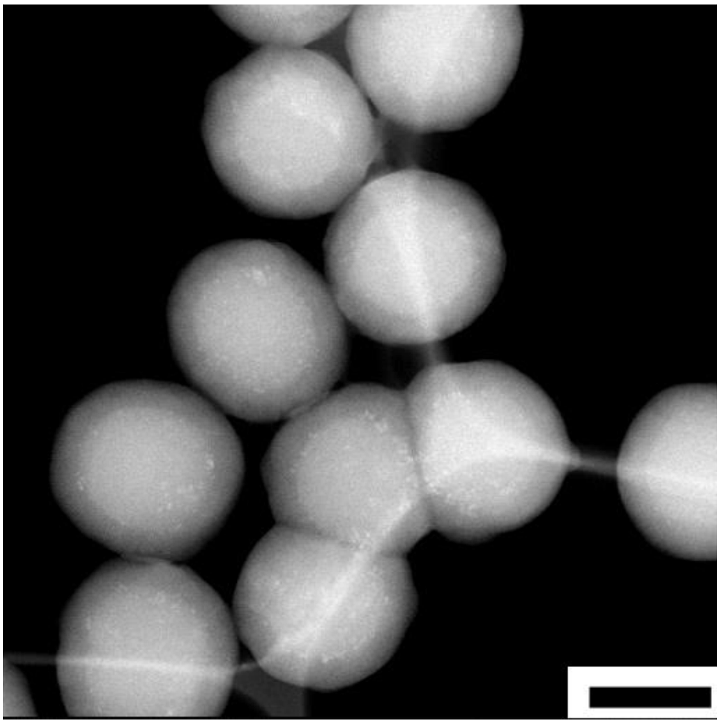

Supplementary Fig. 4. HAADF-STEM image of SiO_2-Nd@SiO_2@mSiO_2-NH_2@SSPI microcarriers. Scale bar = 500 nm.

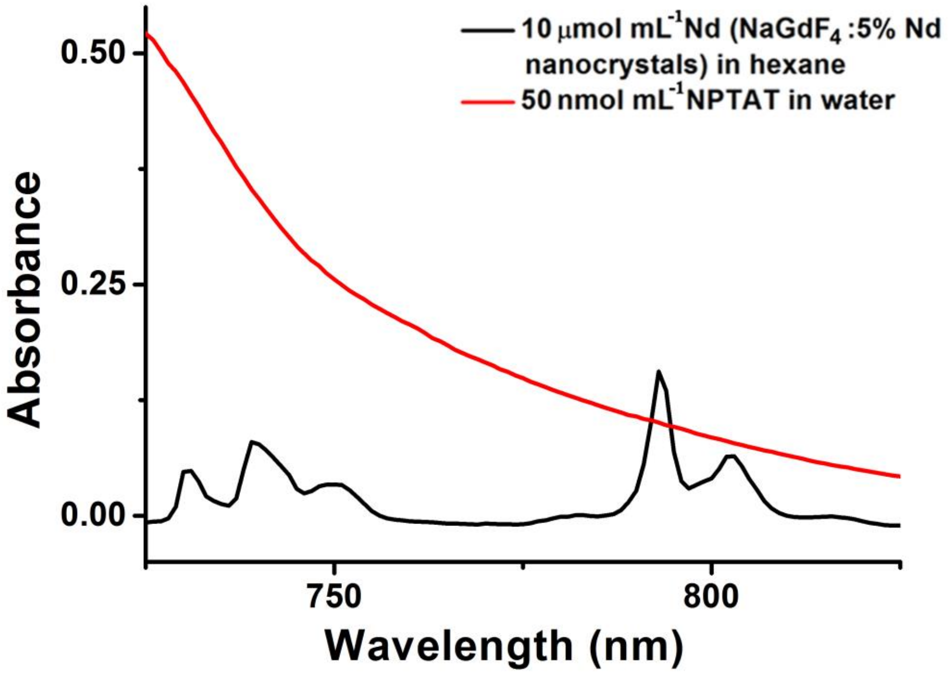

Supplementary Fig. 5. Absorption spectrum of DCNPs from 725 nm to 825 nm.

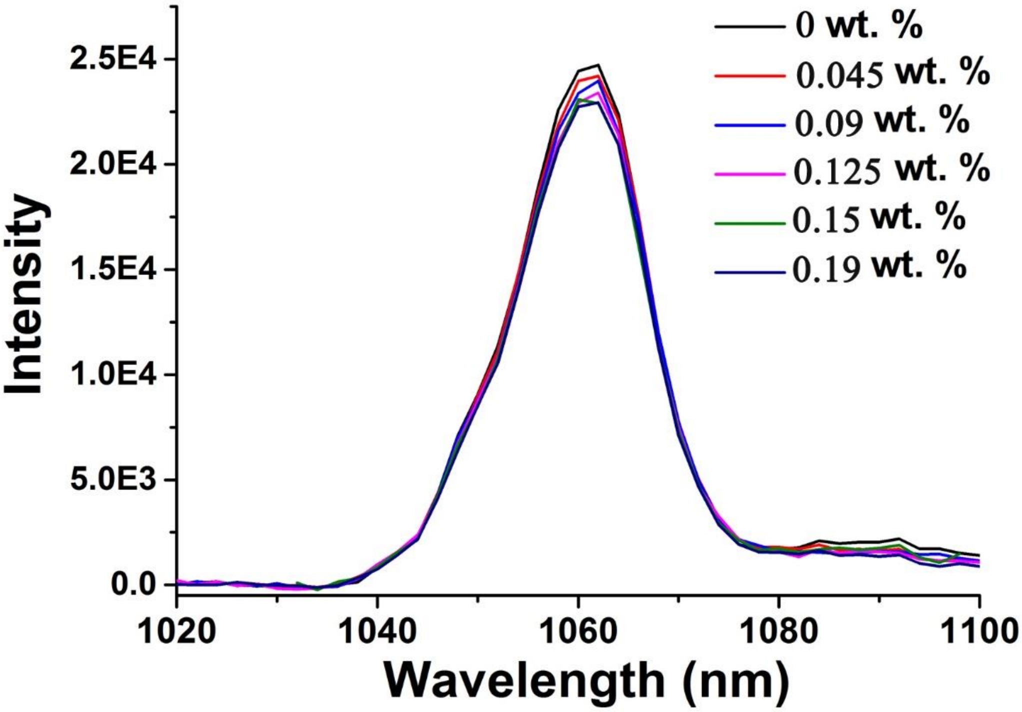

Supplementary Fig. 6. The NIR FL under 808-nm excitation as function of NPTAT loading amount in the microcarriers.

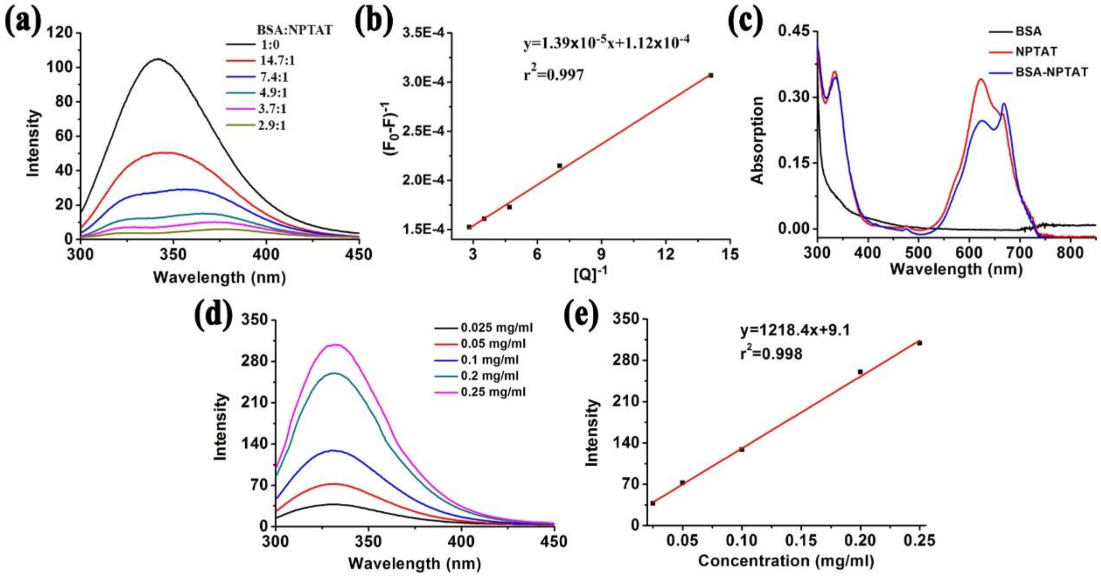

Supplementary Fig. 7. Investigation of interactions between BSA and NPTAT by FL spectroscopy (**a**) Change in FL intensity of BSA (150 nM, excited at 280 nm) in Tris–HCl buffer upon titration with NPTAT at various BSA/NPTAT molar ratio (14.7, 7.4, 4.9, 3.7 and 2.9). The emission intensity of BSA tryptophan residues at 340 nm shows gradual decrease and red-shift upon titration with NPTAT in Tris-HCl buffer. (**b**) Corresponding Lineweaver-Burk plot of (**a**). (**c**) Absorption spectra of BSA (150 nM), NPTAT (20.3 nM) and BSA-NPTAT complex at a BSA/NPTAT molar ratio of 7.4. The absorption band of BSA-NPTAT complex shows slightly red-shift compared with that of pure NPTAT. (**d**) FL intensity of BSA-NPTAT complex (BSA/NPTAT molar ratio of 7.4, excited at 280 nm) as function of complex concentration. (**e**) Linear fit of FL intensity in (**d**), the FL intensity increase proportional with increase of BSA-NPTAT concentration in Tris-HCl buffer. These results indicate the strong affinity in BSA-NPTAT complex.

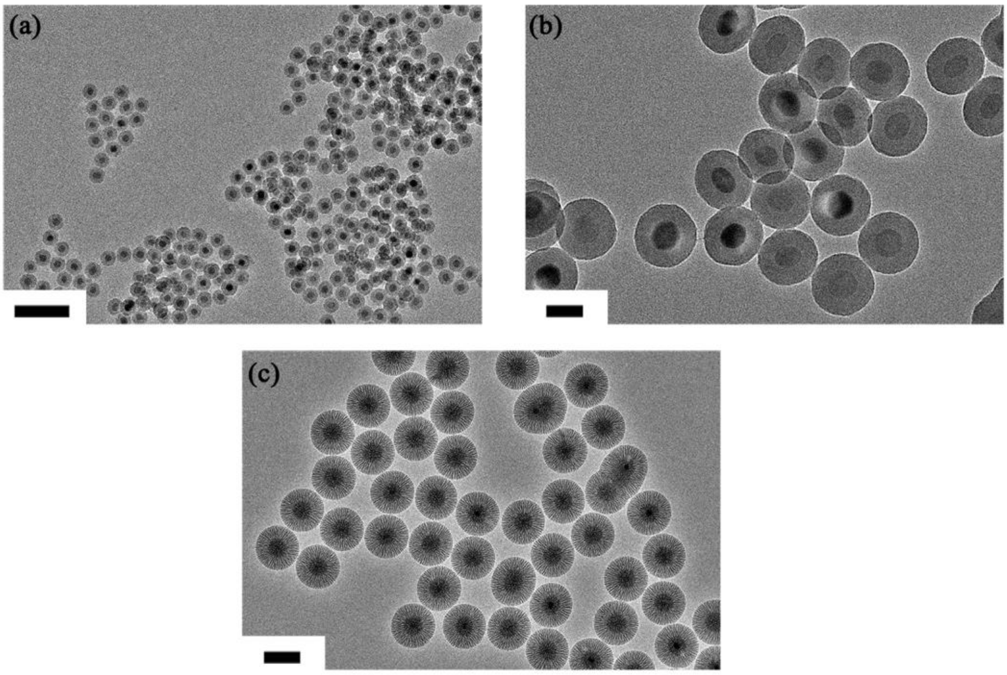

Supplementary Fig. 8. TEM images of microcarriers with a mean size of 25 nm (**a**), 80 nm (**b**), and 300 nm (**c**). Scale bar in (a), (b) and (c) is 100, 50 and 200 nm, respectively.

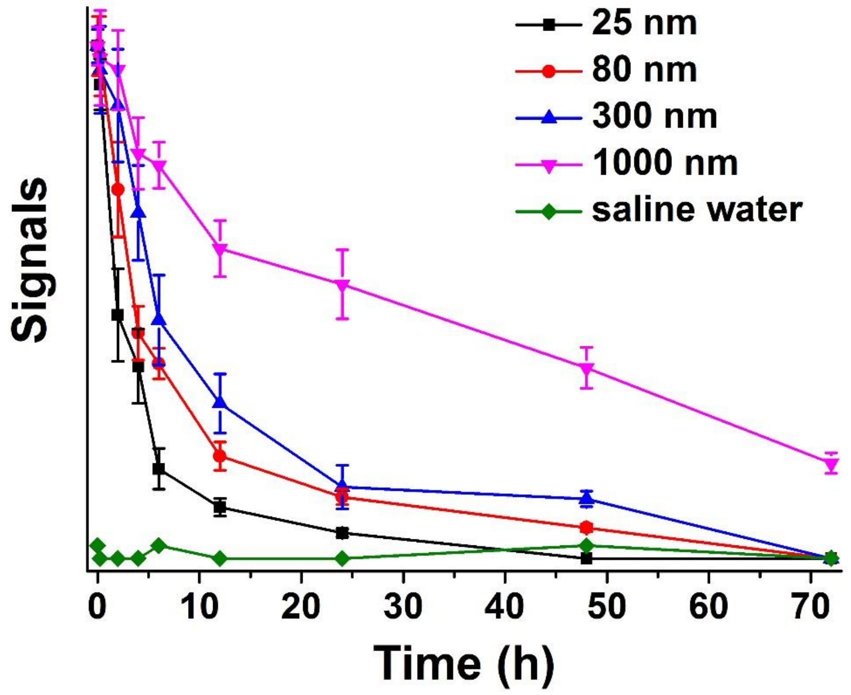

Supplementary Fig. 9. Time dependent normalized NIR-II signal intensity of different sized microcarriers in the GI tract (n = 3 per group).

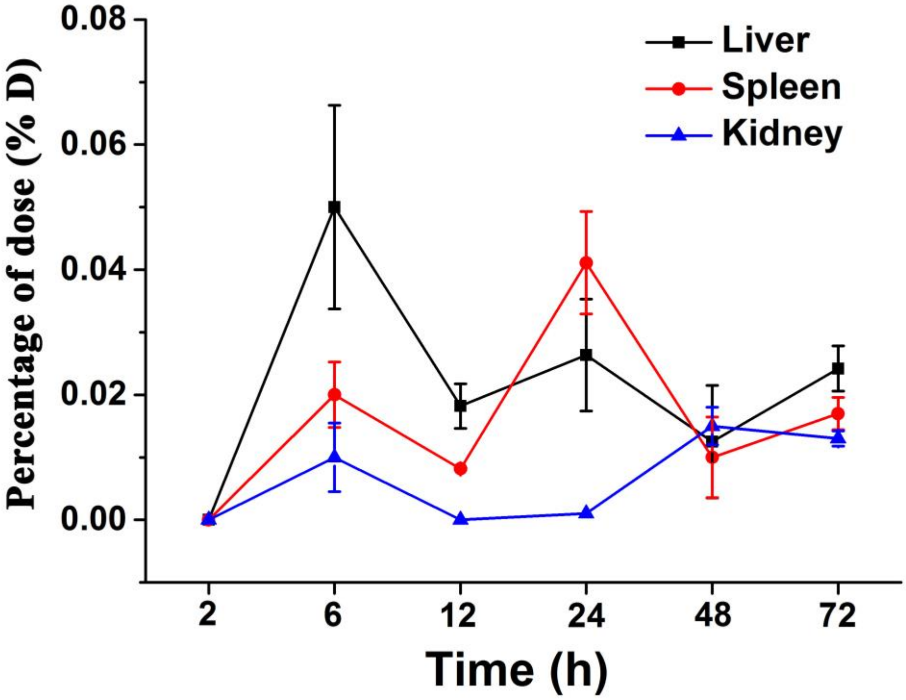

Supplementary Fig. 10. Biodistribution results determined by ICP measurement of 1000 nm microcarriers in visceral organs (liver, spleen and kidney) at different time after oral gavaging. Mean ± s.d. for n = 3.

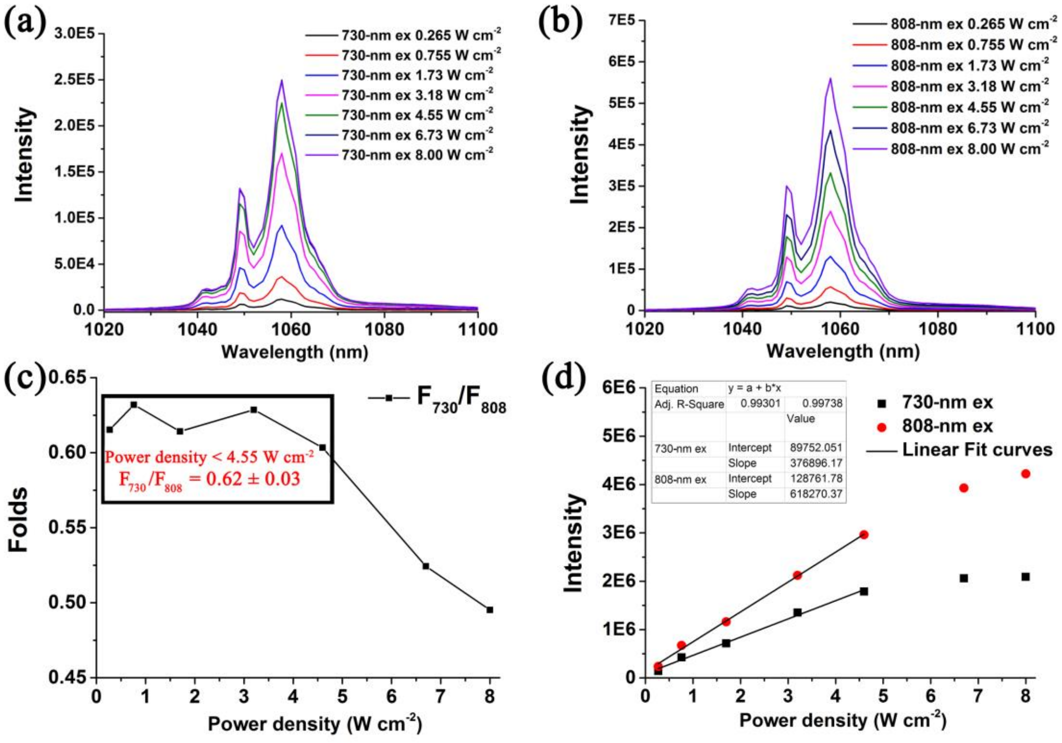

Supplementary Fig. 11. Power dependence of the NIR-II emission intensities of the $NaGdF_4$: 5%Nd@$NaGdF_4$ DCNPs Pump-power-dependent NIR-II FL spectra of $NaGdF_4$:5%Nd@$NaGdF_4$ DCNPs excited by 730 nm (**a**) and 808 nm (**b**). (**c**) Intensity ratio of NIR-II FL (F_{730}/F_{808}) under 730-nm and 808-nm excitation as function of the power density. (**d**) Power density dependence of NIR-II FL of DCNPs.

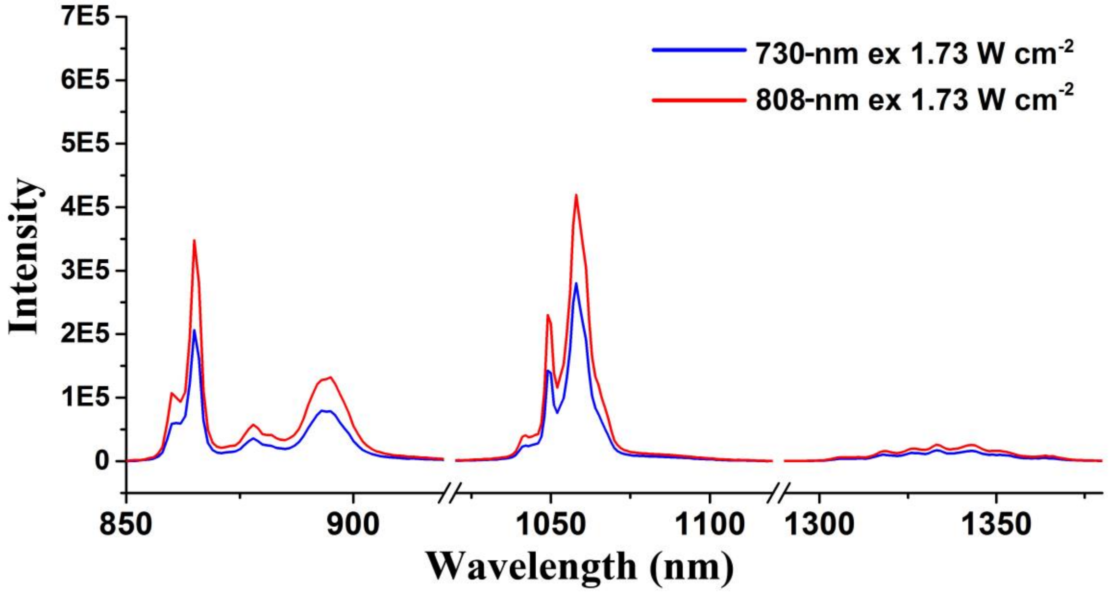

Supplementary Fig. 12. A typical downconversion emission spectrum of $NaGdF_4$:5%Nd@$NaGdF_4$ DCNPs under 730-nm or 808-nm excitation.

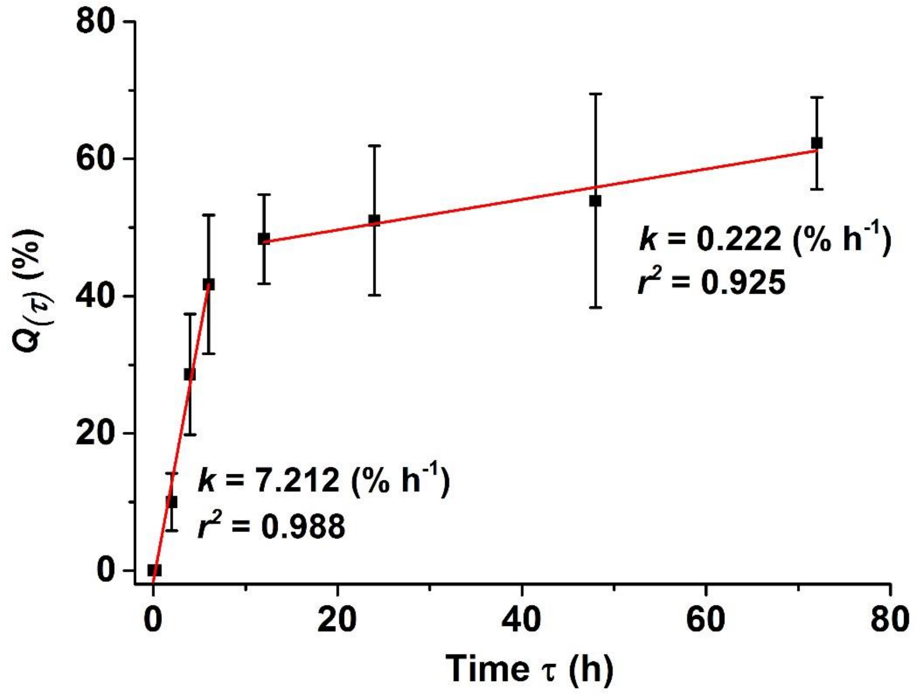

Supplementary Fig. 13. *In vivo* time dependent BSA-NPTAT release percentage data fitted by Eq. (S1).

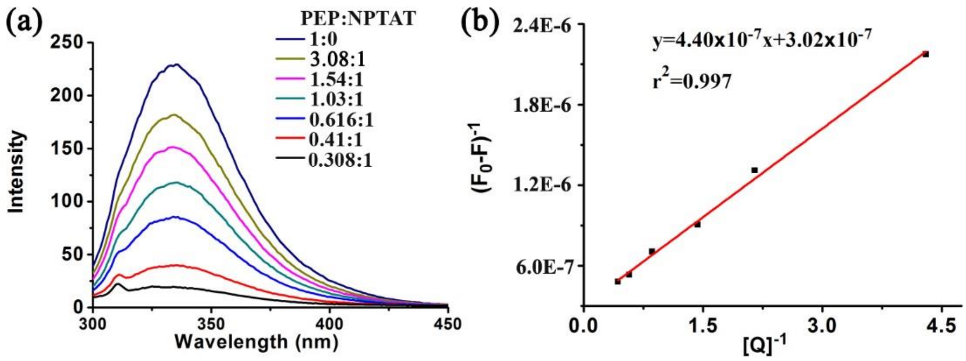

Supplementary Fig. 14. Investigation of interactions between PEP and NPTAT by FL spectroscopy (**a**) Change in FL intensity of PEP (1.23 nM, excited at 280 nm) in Tris-HCl buffer upon titration with NPTAT at various PEP/NPTAT molar ratio (3.08, 1.54, 1.03, 0.616, 0.41 and 0.308). (**b**) Corresponding Lineweaver-Burk plot of (**a**). These results indicate the strong affinity in PEP-NPTAT complex.

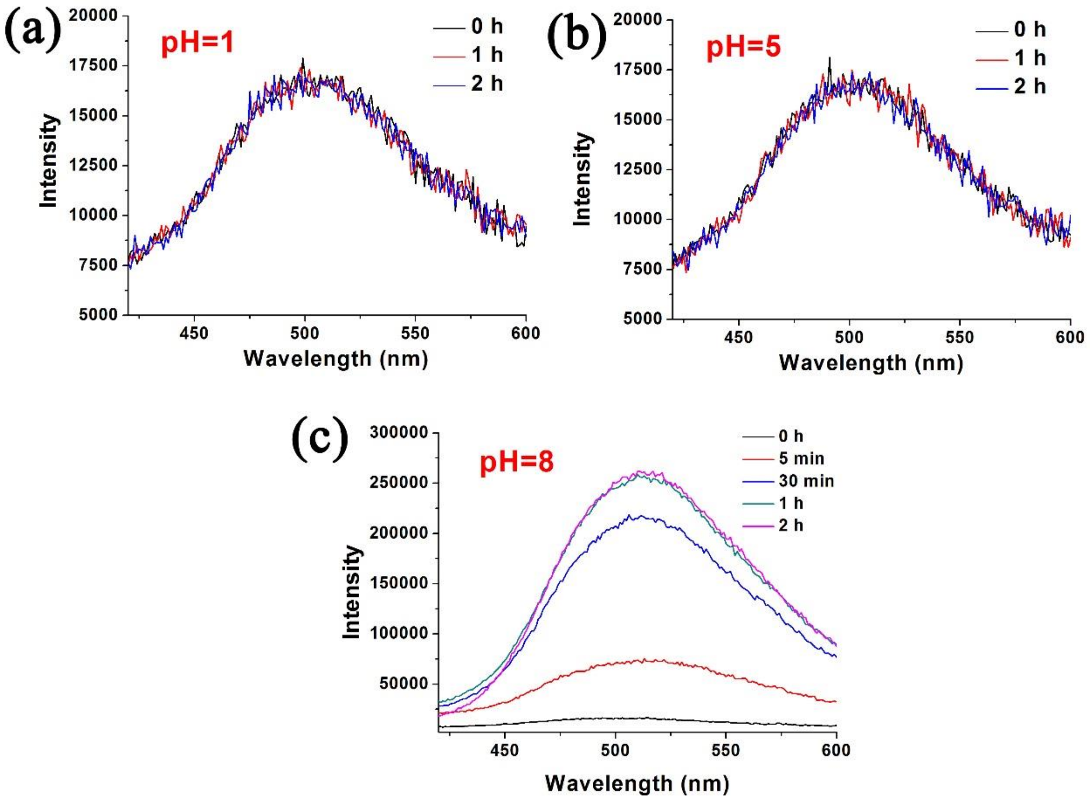

Supplementary Fig. 15. PEP activity examination after *in vitro* release from the microcarriers Time dependent FL intensity of (DABCYL)-LPYPQPK (Glu(EDANS) (20 μg mL^{-1}, excited at 405 nm) upon adding with 0.1 mg microcarriers at various buffer, pH = 1 (**a**), 5 (**b**) and 8 (**c**).

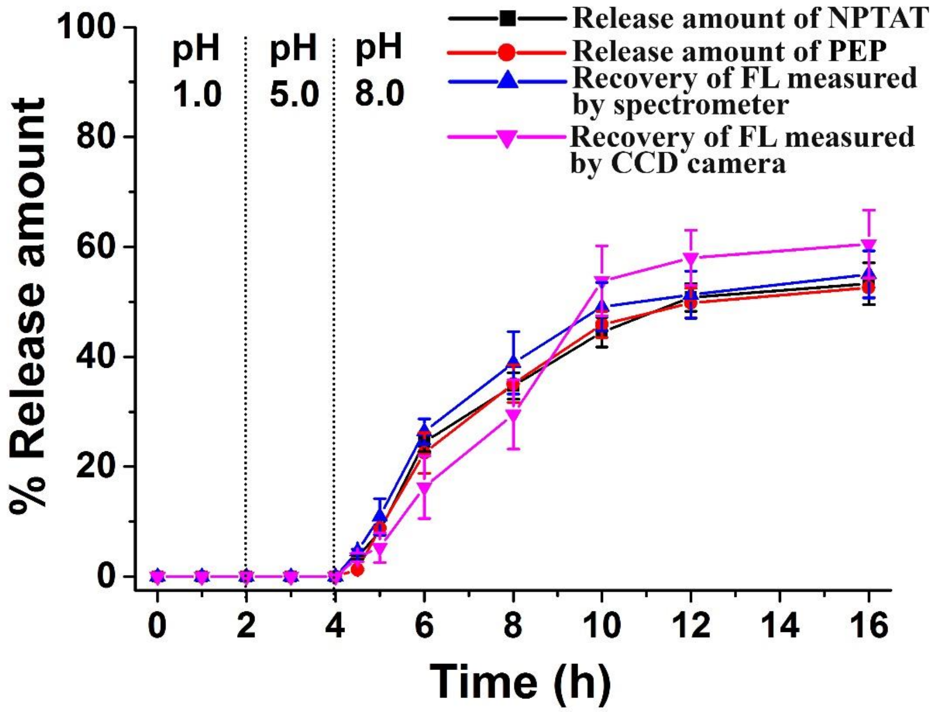

Supplementary Fig. 16. Time-dependent release profiles of PEP-NPTAT loaded microcarriers in simulated GI tract fluids (n = 3 per group).

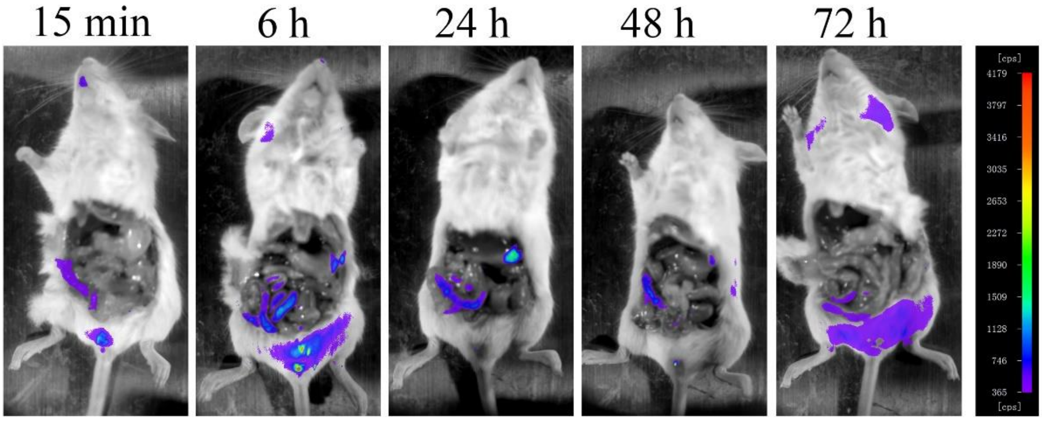

Supplementary Fig. 17. *In vivo* bioimaging of mice after gavaged with peptide probe and PEP-NPTAT loaded microcarriers without SSPI coating. 405 nm was used as excitation source. Representative images for n = 3 per group.

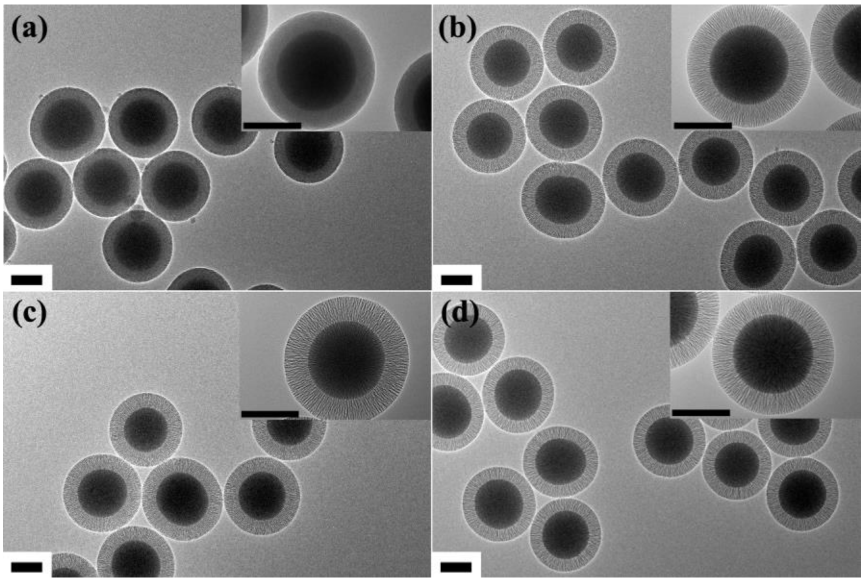

Supplementary Fig. 18. TEM images of microcarriers with pore size of 2.5 nm (a), 7.7 nm (b), 8.9 nm (c) and 12.0 nm (d) The pore size can be easily tuned by changing the reaction condition, such as changing the oil used in the synthesis to octadecene (a), decahydronaphthalene (b) and cyclohexane (c). Microcarriers with pore size of 12 nm (d) were synthesized by reducing the TEOS amount by 25% compared with (c). Scale bar = 200 nm.

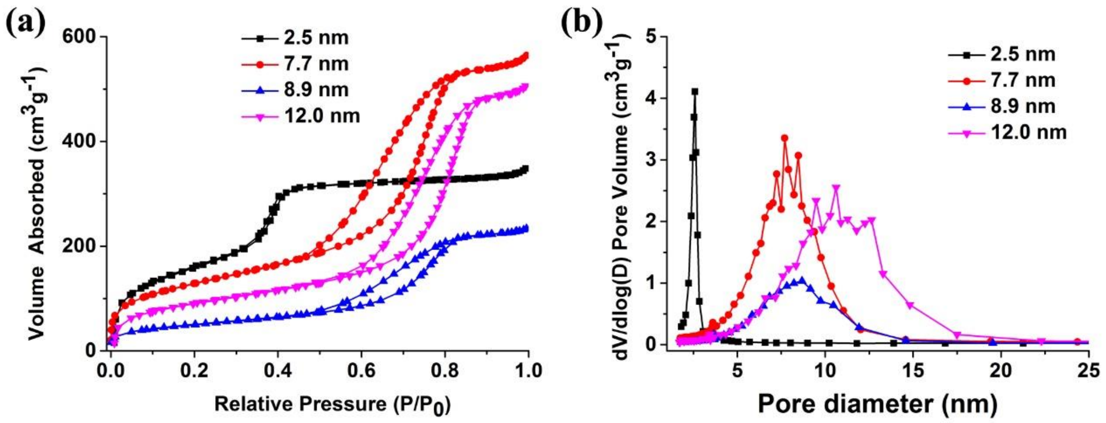

Supplementary Fig. 19. Nitrogen adsorption-desorption isotherms (a) and pore size distribution (b) of different microcarriers, respectively. The corresponding BET surface area was measured to be 586.4, 467.3, 366.8 and 330.1 m^2 g^{-1}, respectively.

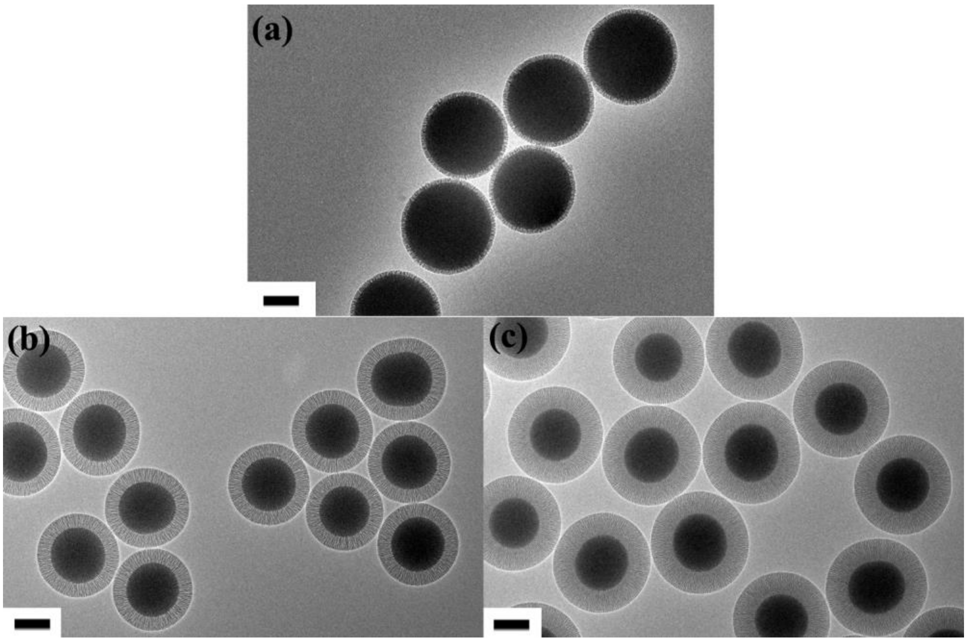

Supplementary Fig. 20. TEM images of microcarriers with pore aspect ratio of 2 : 1 (a), 6 : 1 (b) and 10 : 1 (c). Scale bar = 200 nm.

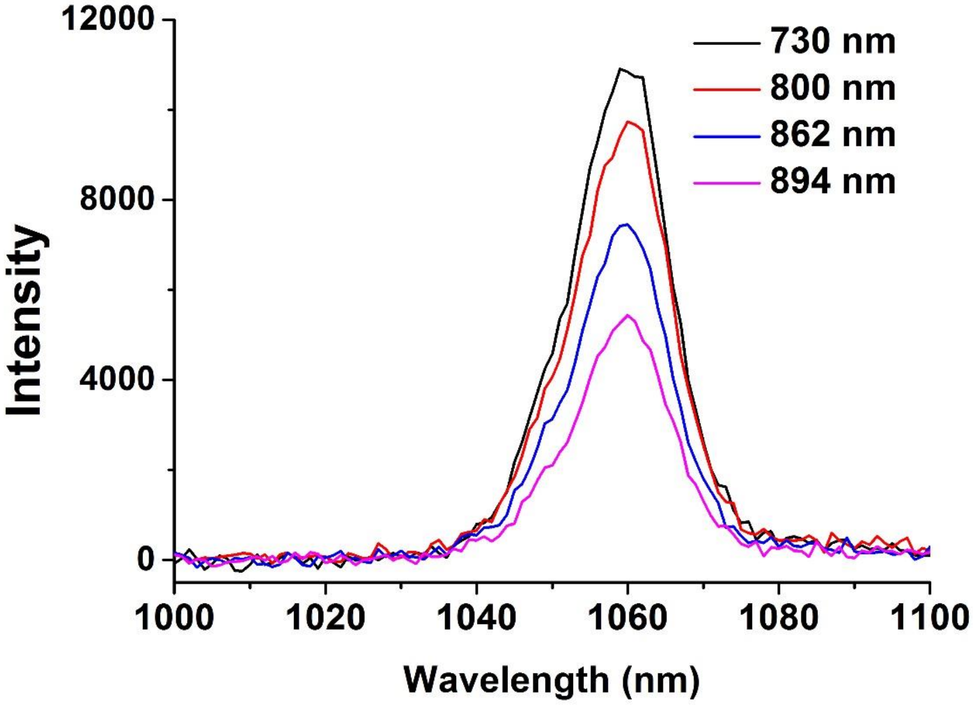

Supplementary Fig. 21. Multi-excitation property of the $NaGdF_4$:5%Nd@$NaGdF_4$ DCNPs The NIR emission spectrum of $NaGdF_4$:5%Nd@$NaGdF_4$ DCNPs (20 mg mL^{-1}, dispersed in cyclohexane) excited by Xenon lamp at different wavelength.

	K_A (M^{-1})	k_q (M^{-1} s^{-1})
BSA-NPTAT	7.2×10^4	7.2×10^{12}
PEP-NPTAT	2.3×10^6	2.3×10^{14}

Supplementary Table 1. The binding constant K_A and bimolecular quenching constant k_q of BSA-NPTAT and PEP-NPTAT complex, calculated by Eq. (3-5)

	25 nm (% D)	80 nm (% D)	300 nm (% D)	1000 nm (% D)
2 h	0.20 ± 0.12	0.12 ± 0.08	0.06 ± 0.03	0.00 ± 0.00
6 h	0.38 ± 0.11	0.23 ± 0.17	0.16 ± 0.05	0.08 ± 0.06
12 h	1.15 ± 0.41	0.58 ± 0.15	0.41 ± 0.18	0.03 ± 0.01
24 h	0.27 ± 0.07	0.20 ± 0.09	0.16 ± 0.07	0.07 ± 0.02
48 h	0.12 ± 0.04	0.07 ± 0.02	0.06 ± 0.01	0.04 ± 0.01
72 h	0.05 ± 0.02	0.05 ± 0.02	0.04 ± 0.01	0.05 ± 0.01

Supplementary Table 2. Percentage of dose (% D) of different sized microcarriers in visceral organs (including liver, spleen and kidney). These data were derived from Fig. 4b-g. Mean ± s.d. for n = 3.

Power density (W cm^{-2})	730-nm excitation			808-nm excitation		
	860 nm (%)	1060 nm (%)	1340 nm (%)	860 nm (%)	1060 nm (%)	1340 nm (%)
0.265	37.09	52.38	10.53	36.85	52.61	10.54
0.755	37.50	52.77	9.73	37.28	53.08	9.64
1.730	36.28	53.91	9.80	38.03	52.44	9.53
3.180	37.41	52.95	9.63	38.79	51.66	9.55
4.550	37.35	53.09	9.56	39.68	50.71	9.62
6.730	37.85	52.46	9.69	40.41	49.79	9.80
8.000	38.21	52.12	9.67	40.40	49.68	9.92

Supplementary Table 3. The proportion of primary emission bands (860, 1060 and 1340 nm) of $NaGdF_4$:5%Nd@$NaGdF_4$ DCNPs excited by 730 or 808-nm at different power density

Time (h)	PEP (mg mL^{-1} h^{-1})	NPTAT (μg mL^{-1} h^{-1})	r_{PEP}/r_{NPTAT}
0	0.0	0.0	-
1	0.0	0.0	-
2	0.0	0.0	-
3	0.0	0.0	-
4	0.0	0.0	-
4.5	8.4	15.3	547
5	7.1	13.2	540
6	5.2	9.9	528
8	2.8	5.5	504
10	1.5	3.1	481
12	0.8	1.7	459
16	0.2	0.5	418

Supplementary Table 4. The release rates of PEP and NPTAT in the *in vitro* release experiment

Time (h)	Cumulative release amount (μg)
0.25	0 ± 0
6	54.8 ± 10.2
24	125.7 ± 11.8
48	127.6 ± 8.5
72	128.2 ± 8.0

Supplementary Table 5. The cumulative release amount of PEP, measured by the Eq. (2)

Supplementary Methods

Materials

Gadolinium (III) chloride anhydrous ($GdCl_3$, 99.99%), neodymium (III) chloride anhydrous ($NdCl_3$, 99.9%), 1-octadecene (ODE, 90%), oleic acid (OA, 90%), sodium trifluoroacetate (Na-TFA, 98%), Nickel (II) phthalocyanine-tetrasulfonic acid tetrasodium (NPTAT), tetraethyl orthosilicate (TEOS), CO-520 surfactant, cetyltrimethylammonium chloride (CTAC) solution (25 wt. % in H_2O), triethanolamine (TEA) and (3-Aminopropyl) triethoxysilane (APTES) were purchased from Sigma-Aldrich. Bull Serum Albumin (BSA, 68 kDa), Sodium hydroxide (NaOH, 96%), ammonium fluoride (NH_4F, 96%), and $NH_3{\cdot}H_2O$ (28 wt. %) were obtained from Beijing Chemical Reagents Co. Ltd. 1,2-distearoyl-sn-glycero-3-phosphoethanolamine-N-[amino(polyethylene glycol)-2000] (DSPE-PEG-NH_2) was purchased from Avanti Polar Lipids. PEP (81 kDa; 30 U mg^{-1}) was obtained from Zedira (Darmstadt, Germany) and used as received. The fluorescence-quenched probe (DABCYL)–LPYPQPK (Glu(EDANS)) was custom-synthesized by Guotai biological (Beijing, China) at 95% purity. Soy protein isolate (SPI) was received as a gift from Prof. Chengzhong Yu, The University of Queensland, Australia. All chemicals were used as received without any further purification.

Synthesis of $NaGdF_4$:5%Nd core DCNPs

The synthesis of the $NaGdF_4$:Nd core DCNPs with a size of ~ 14 nm in this work was similar to the synthesis previously reported by Prasad et *al*.[1] In a typical procedure, 0.95 mmol $GdCl_3$, 0.05 mmol $NdCl_3$, 6.0 mL OA and 15.0 mL ODE were mixed together and heated to 120 °C under vacuum until a clear solution formed. After that, the solution was cooled to room temperature. A solution of 2.5 mmol NaOH and 4.0 mmol NH_4F in 10 mL methanol was added and the mixture was stirred for one hour. The reaction mixture was then heated at 80 °C to remove the methanol. Afterward, the solution was heated to 290 °C and maintained at that temperature for 60 min under a gentle argon flow. Subsequently, the solution was cool down to room temperature and the DCNPs were precipitated, centrifuged and washed twice with ethanol. The DCNPs were finally dispersed in 10 mL of cyclohexane for further use.

Synthesis of $NaGdF_4$:5%Nd@$NaGdF_4$ core/shell DCNPs

Gd-OA (0.10 M) precursor: a mixture of 2.50 mmol $GdCl_3$, 10.0 mL OA, and 15.0 mL ODE were loaded in a reaction container and heated at 140 °C under a vacuum with magnetic stirring for 30 min to remove residual water and oxygen. Then the colorless Gd-OA precursor solution (0.10 M) was obtained.

Na-TFA-OA precursor: A mixture of 4.0 mmol Na-TFA and 10 mL OA was loaded in a container at room temperature under vacuum with magnetic stirring to remove residual water and oxygen. Then the colorless Na-TFA-OA precursor solution (0.40 M) was obtained.

5 mL of the purified $NaGdF_4$:5%Nd core DCNPs solution (~ 0.50 mmol) were mixed with 8.0 mL of OA and 12.0 mL of ODE. The flask was pumped down at 70 °C for 30 min to remove cyclohexane, along with any residual air. Subsequently, the system was switched to Ar flow and the reaction mixture was further heated to 280 °C at a rate of ~ 20 °C min^{-1}. Then pairs of Gd-OA (0.10 M, 1.0 mL) and Na-TFA-OA (0.40 M, 0.50 mL) precursors were alternately introduced by dropwise addition ten times at 280 °C with a 15 minute time interval between each pair of injections. Finally, the obtained $NaGdF_4$:5%Nd@$NaGdF_4$ core/shell DCNPs were washed with ethanol and dispersed in cyclohexane.

Transferring $NaGdF_4$:5%Nd@$NaGdF_4$ DCNPs from hexane solution to aqueous

The phase transfer method used in this work was similar to the method previously reported by Yao et *al*.[2] Typically, 1 mL of oleic acid capped DCNPs in cyclohexane (10 mg mL^{-1}) was mixed with a cyclohexane solution (1 mL) containing 12.5 mg DSPE-PEG-NH_2 in a glass bottle. The glass bottle was left open in a fume hood for two days at room temperature to evaporate the cyclohexane slowly. The resulting mixed film was heated at 75 °C for 5 min to completely remove cyclohexane. Then the film was hydrated with 5 mL water, and the DCNPs became soluble after vigorous sonication and further stirred vigorously at 75 °C for 10 min. The solution was transferred to a microtube and centrifuged and the sediment was discarded to remove possible formation of large aggregates. Excess lipids were purified by ultracentrifugation (15000 rpm, 10 min) and washing.

Preparation of SiO_2 particles

Monodisperse SiO_2 solid particles were prepared by using a modified Stöber method.[3] In a typical process, 4 mL of TEOS was rapidly added into a mixture of 35 mL ethanol, 10 mL H_2O, and

6 mL $NH_3 \cdot H_2O$ (28 wt. %). After continuous stirring at room temperature for two hours, the products were precipitated by adding ethanol. The collected precipitates were washed twice with water and dispersed in 10 mL water for further use.

Preparation of SiO_2-Nd

DCNPs were assembled on the surface of SiO_2 particles by simply mixing them in water. Typically, 2.5 mL of as-synthesized lipid modified DCNPs were added to 2 mL aqueous dispersion of SiO_2 particles. Then, the mixture was gently stirred for 6 hours at 50 °C, followed by washing with water to remove free DCNPs. It is noted that free DCNPs dispersed in the solution can be concentrated and reused again.

Preparation of SiO_2-Nd@SiO_2

In a typical synthesis, the SiO_2-Nd were re-dispersed in a mixed solution containing 6 mL H_2O, 14 mL ethanol and 0.2 mL $NH_3 \cdot H_2O$ with ultrasonication treatment. Subsequently, 0.2 mL of TEOS was added dropwise into the solution. After reaction for 8 h at room temperature, the product was collected by centrifugation and washed with ethanol and water.

Preparation of SiO_2-Nd@SiO_2@mSiO_2

The mesoporous silica shell with large pore size were coated on the surface of SiO_2-Nd@SiO_2 through an oil-water biphase stratification approach by using CTAC as the surfactant template in an alkaline system.[4] In brief, SiO_2-Nd@SiO_2 in was added into a solution containing 48 mL of H_2O, 12 mL of 25 wt. % CTAC/H_2O and 0.18 mL of TEA, followed by the ultrasonication (0.5 h) and stirring(60 °C for 1 h) treatment. Afterwards, 10 mL of TEOS (10 v/v % in cyclohexane) was gently dropped into the upper solution and capped with a stopper. The system was reacted at 60 °C for 12 h in an oil bath with gentle mechanical stirring (200 rpm). The products were collected by centrifugation, and then washed with ethanol and water for several cycles. The template CTAC surfactants and hydrophobic organic phase in the mesopore channels were removed through a solvent extraction method by using 2 wt. % of HCl/ethanol solution refluxed at 60 °C for 10 h. This extraction process was repeated twice to obtain the SiO_2-Nd@SiO_2@mSiO_2.

Preparation of SiO_2-Nd@SiO_2@mSiO_2-NH_2

The amino-modification was achieved via a post-grafting strategy. In a typical process, 15 mL of anhydrous toluene and 100 mg of extracted SiO_2-Nd@SiO_2@mSiO_2 were mixed in a 25 mL round-bottomed flask and stirred vigorously. After formation of a homogeneous suspension, 0.1 mL of APTES was added dropwise to the toluene solution. The reaction was kept at 110 °C with reflux for 20 h. The amino-functionalized products were collected by centrifugation and washed for several times with ethanol to remove the residual reactants and subsequently lyophilized to obtain the dry powder for the next experiments.

Preparation of SiO_2-Nd@SiO_2@mSiO_2-NH_2@SSPI

SSPI can be obtained by succinylating SPI using a modified literature method.[5] Then the carboxylic acid groups on SSPI were activated by EDC (N-(3-Dimethylaminopropyl)-N'-ethylcarbodiimide hydrochloride) and NHS (N-Hydroxysuccinimide). In a typical procedure, 40 mg of SSPI was first dissolved in 10 mL 2-(N-Morpholino) ethanesulfonic acid (MES) buffer (0.1 M, pH 5), 8 mg EDC was added to above solution under stirring, then 8 mg NHS was added, the resulting mixture was stirred for 30 min at room temperature.

Protein-NPTAT composites were prepared before loading. Taking BSA-NPTAT as an example, 30 mg BSA and 0.06 mg NPTAT was dissolved in 6 mL MES buffer (0.1 M, pH 5), followed by 15 min stirring at room temperature. 40 mg SiO_2-Nd@SiO_2@mSiO_2-NH_2 was soaked into this solution, followed by stirring at room temperature for 12 h. The as-prepared BSA-NPTAT loaded SiO_2-Nd@SiO_2@mSiO_2-NH_2 was collected by centrifugation (3000 rpm) and redispersed in 5 mL MES buffer. Then, 10 mL of above SSPI solution (4 mg mL^{-1} in 0.1 M MES buffer) was added quickly, and the obtained turbid solution was further stirred for 2 hours at room temperature. The resulting SiO_2-Nd@SiO_2@mSiO_2-NH_2@SSPI were finally centrifuged at 3000 rpm, washed several times with MES buffer to remove unreacted SSPI followed by lyophilizing.

Similar procedures were done on the PEP-NPTAT loaded SiO_2-Nd@SiO_2@mSiO_2-NH_2@SSPI.

Preparation of 25, 80 and 300 nm microcarriers

Both 25 and 80 nm microcarriers were synthesized by a reversed-phase microemulsion method. In a typical procedure to synthesize 25 nm microcarrier, 30 mg $NaGdF_4$:5%Nd core DCNPs (~ 10

nm) were added to a mixing solution of 2.5 mL CO-520, 20 mL cyclohexane and 0.3 mL $NH_3 \cdot H_2O$ (28 wt. %), and the container was sealed and sonicated for 15 min until a transparent reversed-phase microemulsion was formed. Then, 0.1 mL TEOS was added into the solution. After continuous stirring at room temperature at the speed of 450 rpm for two days, products were precipitated by adding ethanol. The collected precipitate was washed with ethanol twice and dispersed in saline water. To synthesize 80 nm microcarrier, the procedure was similar that of the 25 nm microcarrier, except that $NaGdF_4$:5%Nd@$NaGdF_4$ core/shell DCNPs (~ 40 nm) were used, instead of $NaGdF_4$: 5%Nd core DCNPs.

300 nm microcarrier were synthesized by coating mesoporous silica shell on the 80 nm microcarrier. The procedure was similar with the preparation of SiO_2-Nd@SiO_2@mSiO_2, except that 80 nm microcarrier were used as seeds, instead of SiO_2-Nd@SiO_2.

All of these particles were modified with amino group, followed by SSPI grafting. The synthesis procedure was similar to the preparation of SiO_2-Nd@SiO_2@mSiO_2-NH_2@SSPI.

Preparation of simulated body fluids

Simulated gastric fluid: 2.0 g NaCl was dissolved to 1000 mL H_2O, then the pH was adjusted to 1.0 using 1 M HCl.

Simulated duodenum fluid: 1.36 g KH2PO4 and 0.304 g NaOH were dissolved to 200 mL H_2O, then the pH was adjusted to 5.0.

Simulated intestine fluid: The simulated intestine fluid was obtained by adjusting the pH of simulated duodenum fluid to 8.0 using 1 M NaOH aqueous solution.

Power dependence study of the NIR-II emission intensities of DCNPs

By increasing the pump power density up to 4.55 W cm^{-2}, both 730-nm excited and 808-nm excited NIR-II emission intensities increased proportional to the increase of power density (Supplementary Fig. 11a,b). Significantly, when the power density was below this threshold (4.55 W cm^{-2}), the NIR-II emission intensity under 730-nm was about 62% of that obtained under 808-nm excitation (F_{730}/F_{808}) (Supplementary Fig. 11c). Beyond this threshold, the NIR-II emission intensity under 730-nm excitation quickly reached a plateau while NIR-II emission intensity under 808-nm excitation maintained increasing, leading to decrease of F_{730}/F_{808} (Supplementary Fig.

11c,d). Since safe-exposure limit of laser for small animals (0.23 W cm^{-2} at 730 nm and 0.33 W cm^{-2} at 808 nm)[6], the laser power density used in the present work was much lower than 4.55 W cm^{-2}, which means that the F_{730}/F_{830} would be a constant (~ 0.62) in the experiment.

***In vivo* pharmacokinetics investigation of BSA by using radioisotopic tracing method**[7]

1000 nm microcarriers were used in the following experiment.

Labeling and Purification of ^{125}I-BSA

BSA was radiolabeled with ^{125}I-Na using iodogen method. Labeled BSA was separated using Sephadex G-25 (0.4 × 8 cm) column and drenched with 0.01 M phosphate-buffered saline (pH 7.4). The elution fractions were collected and free ^{125}I in the fraction was removed using ultrafiltration tubes with a molecular weight cutoff of 1 kDa. The purity of ^{125}I-BSA was assessed using HPLC method and SDS-PAGE. The radioactivity was measured using γ-counter. The resulting radiochemical purity and activity concentration of the resulting ^{125}I-BSA was measured to be 98% and 1.54 μCi $μg^{-1}$.

Fabrication of ^{125}I-BSA loaded microcarriers

0.7 mg ^{125}I-BSA was mixed with 4.3 mg un-labeled BSA in 2 mL MES buffer (0.1 M, pH 5), followed by 15 min stirring at room temperature. 12 mg SiO_2-Nd@SiO_2@mSiO_2-NH_2 was soaked into this solution, followed by stirring at room temperature for 1 h. The as-prepared ^{125}I-BSA-NPTAT loaded SiO_2-Nd@SiO_2@mSiO_2-NH_2 was collected by centrifugation (3000 rpm) and redispersed in 2 mL MES buffer. Then, 3 mL of above SSPI solution (4 mg mL^{-1} in 0.1 M MES buffer) was added quickly, and the obtained turbid solution was further stirred for 1 hours at room temperature. The resulting microcarriers were finally centrifuged at 3000 rpm and washed several times with MES buffer to remove unreacted SSPI. By measuring the radiation dose of the supernatants, loading efficiency was calculated to be 18.2%.

***In vivo* pharmacokinetics investigation**

Kunming mice (5-6 weeks, female) were fasted for 12 h before experiment. Then the animals were randomized into four groups with n = 3 per group. Mice in all groups received a single dosage of ^{125}I-BSA loaded microcarriers (50 mg kg^{-1}) via oral gavaging. The animals were sacrificed at 2, 6, 12 or 72 h after gavaging. Blood, excretion (including urine and feces) and tissues (including stomach, intestine, liver and kidney) were collected and weighed. Before measuring radioactivity,

trichloroacetic acid precipitation method were used to remove free $^{125}I^-$ ions and fragmented BSA.

Investigation of *in vivo* drug release kinetics

For the first 12 hours of drug release *in vivo*, which corresponds to about 50% of released drug, the release rates differ greatly with those after 12 h, therefore, the release data before 12 h and after 12 h were fitted separately. Taking BSA-NPTAT loaded microcarriers as an example, the release percentage data were fitted to the following equation:

$$Q_{(\tau)} = kt^n \tag{S1}$$

Where $Q_{(\tau)}$ is the release percentage of the BSA-NPTAT at time τ (Fig. 6c), k is release rate constant, and n is the release exponent, indicative of the mechanism of release.

Supplementary Fig. 13 showed that release percentage data before 12 h and after 12 h both behave as one-order release system (release exponent $n = 1$), except the release rate constant k before 12 h (7.212 (% h^{-1})) is much larger than the latter (0.222 (% h^{-1})).

Characterization

Transmission electron microscopy (TEM), high-resolution transmission electron microscopy (HRTEM), selected area electron diffraction (SAED) and electron energy loss spectroscopy (EELS) observations were performed with a JEM-2100F transmission electron microscope with an accelerating voltage of 200 kV equipped with a post-column Gatan imaging filter (GIF-Tri-dium). Visible FL spectra were recorded on Edinburgh Fluorescence Spectrometer FLS980 instrument with Xenon lamp as excitation source. While NIR FL spectra were also recorded on this instrument, but excitation source were using external 808-nm or 730-nm semiconductor laser (Changchun New Industries Optoelectronics Tech. Co., Ltd.), instead of the Xenon source in the spectrophotometer (Unless otherwise specified, all spectra were collected under identical experimental conditions). The UV/Vis spectra were recorded on Lambda 35 PerkinElmer. Optical luminescence imaging of the mice organs after dissection was performed with LB983 NightOWL II (Berthold Technologies GmbH & Co.KG, Germany). *In vivo* NIR images was taken using NIR vana CCD camera (Princeton Instruments Inc.). The distribution of microcarriers were determined by measuring silicon content in different organs by inductively coupled plasma atomic emission spectrometry (ICP) using an IRIS Advantage Duo ER/S (Thermo Fisher Scientific).

Supplementary References

1. Chen, G., et al. Core/shell NaGdF4:Nd3+/NaGdF4 nanocrystals with efficient near-infrared to near-infrared downconversion photoluminescence for bioimaging applications. *ACS Nano* **6**, 2969-2977 (2012).
2. Yao, C., et al. Highly biocompatible zwitterionic phospholipids coated upconversion nanoparticles for efficient bioimaging. *Anal. Chem.* **86**, 9749-9757 (2014).
3. Stober, W., Fink, A. & Bohn, E. Controlled growth of monodisperse silica spheres in micron size range. *J. Colloid Interface Sci.* **26**, 62-69 (1968).
4. Shen, D., et al. Biphase stratification approach to three-dimensional dendritic biodegradable mesoporous silica nanospheres. *Nano Lett.* **14**, 923-932 (2014).
5. Popat, A., et al. Programmable drug release using bioresponsive mesoporous silica nanoparticles for site-specific oral drug delivery. *Chem. Commun.* **50**, 5547-5550 (2014).
6. Matthes, R., et al. Revision of guidelines on limits of exposure to laser radiation of wavelengths between 400 nm and 1.4 µm. *Health Phys.* **79**, 431-440 (2000).
7. Tang, X., et al. Pharmacokinetics and pharmacology of hirulog-like peptide. *J. Cardiovasc. Pharmacol.* **50**, 406-410 (2007).
